# Opportunities to Address Specialty Care Deserts and the Digital Divide through the Veterans Health Administration’s Telehealth Hub-and-Spoke Cardiology Clinic: Retrospective Cohort Study

**DOI:** 10.2196/53932

**Published:** 2024-11-28

**Authors:** Rebecca Lauren Tisdale, Colin Purmal, Neil Kalwani, Alexander Sandhu, Paul Heidenreich, Donna Zulman, Tanvir Hussain

**Affiliations:** 1 Center for Innovation to Implementation (Ci2i) Health Services Research VA Palo Alto Health Care System Menlo Park, CA United States; 2 Department of Medicine School of Medicine Stanford University Stanford, CA United States; 3 VA San Francisco Healthcare System San Francisco, CA United States; 4 Department of Medicine School of Medicine University of California San Francisco, CA United States

**Keywords:** telehealth, specialty care, cardiovascular disease, telemedicine, cardiology, veterans, low income, digital divide, access, Veterans Health Administration, VA, VHA, rural, clinical resource hub, CRH

## Abstract

**Background:**

To address geographic barriers to specialty care access for services such as cardiology, the Veterans Health Administration (VA) has implemented a novel, regionalized telehealth care hub. The Clinical Resource Hub (CRH) model extends care, including cardiology services, to individuals in low-access communities across the region. Little is known, however, about the reach of such programs.

**Objective:**

This study aimed to describe the initial CRH program implementation in terms of growth in users and clinical encounters, as well as the association between user characteristics and the use of CRH cardiology care, in VA’s Sierra Pacific region (Northern California, Nevada, and the Pacific Islands).

**Methods:**

We compared patients who used CRH cardiology services (CRH users) to those using non-CRH cardiology services (CRH nonusers) in the Sierra Pacific region between July 15, 2021, and March 31, 2023. After characterizing changes in the numbers of CRH users and nonusers and clinical encounters over the study period, we used multivariable logistic regression to estimate the association between patient-level factors and the odds of being a CRH user.

**Results:**

There were 804 CRH users over the study period, with 1961 CRH encounters concentrated at 3 main CRH sites. The CRH program comprised a minority of cardiology users and encounters in the region, with 19,583 CRH nonusers with 83,489 encounters. The numbers of CRH patients and encounters both increased at a steady-to-increasing rate over the study period, with increases of 37% (n=292 vs n=213) in users and 64% (n=584 vs n=356) in encounters in the first quarter of 2023 compared with the last quarter of 2022. Among CRH users, 8.3% (67/804) were female and 41.4% (333/804) were aged ≥75 years, compared with 4.3% (840/19,583) and 49% (9600/19,583), respectively, among CRH nonusers. The proportions of rural (users: 205/804, 25.5%; nonusers: 4936/19,583, 25.2%), highly disabled (users: 387/804, 48.1%; nonusers: 9246/19,583, 47.2%), and low-income (users: 165/804, 20.5%; nonusers: 3941/19,583, 20.1%) veterans in both groups were similar. In multivariable logistic models, adjusted odds ratios of using CRH were higher for female veterans (1.70, 95% CI 1.29-2.24) and lower for older veterans (aged ≥75 years; 0.33, 95% CI 0.23-0.47). Rural veterans also had a higher adjusted odds ratio of using CRH (1.19, 95% CI 1.00-1.42; *P*=.046).

**Conclusions:**

The VA’s Sierra Pacific CRH cardiology program grew substantially in its first 2 years of operation, serving disproportionately more female and rural veterans and similar proportions of highly disabled and low-income veterans compared to conventional VA care. This model appears to be effective for overcoming specialty care access barriers for certain individuals, although targeted efforts may be required to reach older veterans. While this study focuses on a single region, specialty, and health care system, lessons from implementing regionalized telehealth hub models may be applicable to other settings.

## Introduction

Access to specialty care varies widely across US geographic regions, a pattern that poses problems for the delivery of cardiology care within the Veterans Health Administration (VA) [1-3]. Given the high prevalence of cardiovascular disease and associated morbidity and mortality among veterans [4], maintaining access to cardiology care is essential. As there are unique and disproportionately high risks of dual use of VA and community care for veterans with cardiovascular disease [5], maintaining access to VA-based cardiology care is a particular priority. Beyond the VA, patient subpopulations across the general United States, such as women [6] and those living in rural areas [7,8], similarly face barriers to accessing specialty cardiology care.

Telehealth (new or follow-up patient visits, delivered by phone or video) expanded significantly during the COVID-19 pandemic in cardiology [9] across the VA [10] and other health care systems. In this post–public health emergency phase of the pandemic, patient familiarity with these modalities of care provides opportunities for new ways of using telehealth, including improving access to specialty care in nonemergency settings.

The VA’s Clinical Resource Hub (CRH) model of care offers mostly telehealth care to individuals in low-access regions. The CRH cardiology program was first implemented in July 2021 in VA’s Sierra Pacific region, which serves Northern California, Nevada, and the Pacific Islands (Textbox 1).

**Veterans Health Administration (VA) Clinical Resource Hub (CRH) program.**

Given the concentration of specialists in urban settings, the CRH model leverages specialist availability at an urban site to serve as the regional hub, where these providers extend their services (largely) through telehealth to distant sites. The Palo Alto VA site serves as the hub for specialty care, including cardiology, for the VA’s CRH program in the Sierra Pacific region. Spoke site–specific contracts known as telehealth service agreements detail the relationship between the Palo Alto–based CRH clinical team and other individual sites; the spoke sites implementing CRH cardiology in 2021-2023 in this region were VA’s Sierra Nevada (Reno), Southern Nevada (Las Vegas), and Northern California (Sacramento) sites. The services available through CRH at each spoke site (eg, which subspecialty clinics, such as heart failure or women’s health cardiology) depend on the site’s needs and service gaps, and the program employs physicians, nurses, pharmacists, and administrative staff.

While the initial implementation and usage of the CRH model has been described in primary and mental health [11,12] and for certain specialties [13,14], expansion of the program for cardiology specialty care has yet to be characterized. In this study, we have analyzed the initial implementation of the VA Sierra Pacific Region CRH cardiology program, describing the sociodemographic characteristics of users, program growth, and different modalities of care across the program.

## Methods

### Overview

This analysis was conducted as a clinical operations quality improvement project through the VA Sierra Pacific Region CRH leadership team. Using VA’s Corporate Data Warehouse, we constructed a cohort of all patients with at least 1 evaluation and management encounter (visit) in any CRH or conventional VA-based cardiology clinic in VA’s Sierra Pacific region (encompassing Northern California, Nevada, and the Pacific Islands) between July 15, 2021, when the first CRH site was first implemented in this region, and March 31, 2023.

### Patient Characteristics

Patient characteristics were extracted for the first available data during the study period (from July 15, 2021, to March 31, 2023). We included the following patient-level sociodemographic data: age, sex, race, and ethnicity (American Indian or Alaska Native, Asian, Black or African American, Native Hawaiian or Pacific Islander, White, or unknown), rurality (highly rural, rural, or urban), and home site for receiving VA care. We also included VA enrollment priority as a proxy for social need, based on the VA’s enrollment priority classification system [15]; this program categorizes VA patients according to military service–related disability and income and influences whether patients pay copays and what services they can access within VA. As in previous published literature [9,10], we condensed these into four enrollment priority categories: (1) high disability, corresponding to enrollment priority groups 1 and 4; (2) low-moderate disability, including priority groups 2, 3, and 6; (3) low-income, including priority group 5; and (4) no disability nor low-income status, wherein patients pay copays for VA care, including priority groups 7-8. Due to the hierarchical nature of these groups, veterans assigned to high- or low-moderate disability groups may also be low income.

We captured cardiovascular diagnoses based on primary diagnoses at cardiology visits, grouping these into several categories representing the most commonly-coded primary diagnoses: heart failure, ischemic heart disease, valvular heart disease, and atrial fibrillation or flutter. *International Classification of Diseases, 10th Revision* (*ICD-10*) codes are shown in Table S1 in Multimedia Appendix 1.

### Encounter Characteristics

We captured the primary diagnosis group assigned to a given encounter as described above, as well as the encounter hub and spoke sites. We also collected data on encounter modality: phone, video (either direct to the patient’s home through the VA’s video platform, or from the cardiology team to a local clinic), or in person.

### Statistical Analysis

For our descriptive analysis, we calculated the proportions of CRH users and nonusers with a given sociodemographic characteristic for all the characteristics outlined above. We calculated standardized mean differences with Cramer V for categorical variables and through Cohen *d* for continuous variables and binary categorical variables to contextualize differences between groups; standardized mean differences are more informative than *P* values when analyses have a large sample size, as even small differences between groups are statistically significant.

We constructed a logistic regression model with the primary outcome of adjusted odds of being a CRH user, with all the covariates outlined above as well as a fixed effect for the patient’s assigned primary care site and robust SE. Because the VA’s expansion of telehealth has prioritized video visits [16], we then constructed a separate logistic regression model of adjusted odds of being a video care user with every use of CRH as an additional covariate. All analyses were performed in Stata 18 (StataCorp LLC) and used a *P* value level of .05 to assess statistical significance. For all models, we treated missing data for a given characteristic as a separate category. The proportion of missing data was low (highest for ethnicity: 1231/20,387, 6%) for all characteristics except race, where 10.1% (2066/20,387) of patients were missing race information.

### Ethical Considerations

This analysis was carried out as nonresearch quality improvement by VA program office partners in the VA Veterans Integrated Service Network 21 CRH operations team and was thus considered nonhuman participants’ research. Therefore, it was exempt from institutional review board approval. Informed consent was waived, and patients were not compensated as data were collected in the course of normal clinical operations and used for quality improvement. Data analysis took place on a secure server to ensure privacy and confidentiality protection.

## Results

### Patients

There were 804 CRH users over the study period with a total of 4315 ambulatory cardiology encounters, 1961 of which were CRH encounters. Just over half of CRH users (403/804, 50.1%) had non-CRH cardiology encounters in addition to CRH encounters. In addition, there were 19,583 CRH nonusers with 83,489 ambulatory encounters, meaning CRH users comprised 3.9% (804/20,387) of the total patients using ambulatory cardiology services in the region over the study period.

Among CRH users, 8.3% (67/804) were female and 41.4% (333/804) were aged ≥75 years, compared with 4.3% (840/19,583) and 49% (9600/19,583), respectively, among CRH nonusers (Table 1). Similar proportions in both groups were rural or highly rural (CRH users: 205/804, 25.5%; CRH nonusers: 4936/19,583, 25.2%), highly disabled according to VA enrollment categorization (CRH users: 387/804, 48.1%; CRH nonusers: 9246/19,583, 47.2%), and low-income (CRH users: 165/804, 20.5%; CRH nonusers: 3941/19,583, 20.1%). Somewhat higher proportions of CRH users had diagnoses of atrial fibrillation or flutter (CRH users: 316/804, 39.3% and CRH nonusers: 6300/19,583, 32.2%), heart failure (CRH users: 149/804, 18.5% and CRH nonusers: 3405/19,583, 17.4%), and valvular heart disease (CRH users: 158/804, 19.7% and CRH nonusers: 2530/19,583, 12.9%); however, the proportion of CRH nonusers with a diagnosis of ischemic heart disease was higher than for CRH users (CRH nonusers: 8137/19,583, 41.6% and CRH users: 256/19,583, 31.8%). Similar proportions of CRH users and nonusers (CRH users: 205/804, 25.5% and CRH nonusers: 5393/19,583, 27.5%) had none of these diagnoses, that is, had a primary diagnosis other than atrial fibrillation or flutter, heart failure, valvular heart disease, or ischemic heart disease.

**Figure 1:**
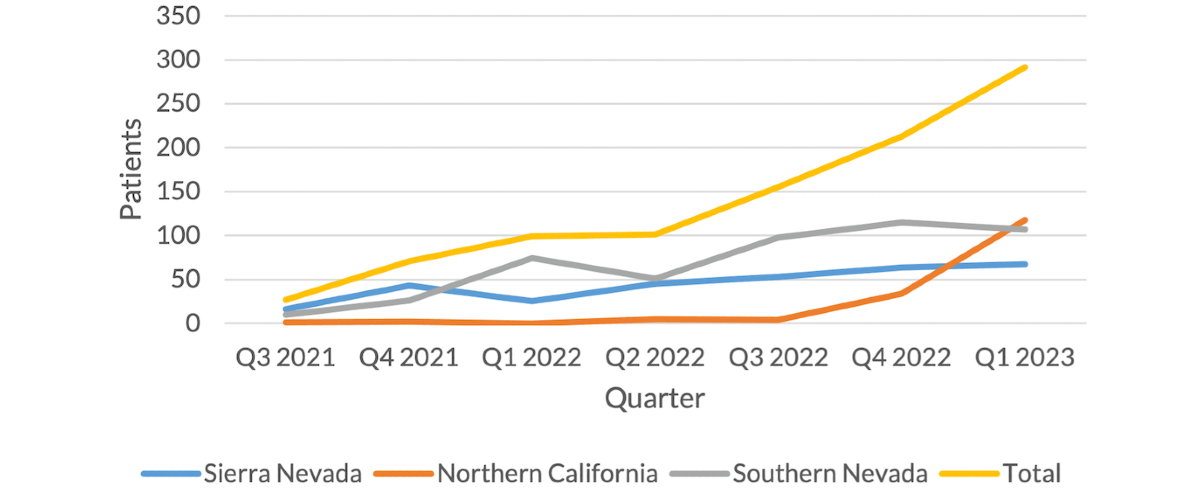
Clinical Resource Hub patients seen at implementing sites over time (total unique patients: n=804; unique patients across sites depicted: n=688). Q: quarter.

Figure 1 shows the number of CRH patients seen quarterly over time at the 3 sites with the most CRH encounters (Sierra Nevada, or Reno; Southern Nevada, or Las Vegas; and Northern California, or Sacramento). Quarterly patients generally increased over time (Figure 1), with some sites’ growth rates picking up more abruptly (eg, Northern California) and others demonstrating a steadier increase (Sierra Nevada, Southern Nevada). Initial CRH encounters for these sites were July 29, 2021 (Southern Nevada); September 8, 2021 (Sierra Nevada); and September 21, 2021 (Northern California). Total unique patients increased over the previous quarter by 37% (n=292 vs n=213) in both the fourth quarter of 2022 and the first quarter of 2023 compared with the previous quarters. This trend was largely driven by rapid growth at the Northern California site, where patients more than tripled between the last quarter of 2022 and the first quarter of 2023.

**Table 1:** Characteristics of cardiology patients, clinical resource hub users (n=804), and nonusers (n=19,583).

			CRH^a^ users (n=804)		CRH nonusers (n=19,583)		Standard mean difference^b^
**Used video care^c^** **, mean (SD)**			438 (54.5)		1633 (8.3)		0.29
**Age (years), mean (SD)**			69.5 (12.8)		72.7 (11.1)		–0.05
**Age group (years), n (%)**							0.25
	18-44	50 (6.2)		515 (2.6)		—^d^	
	45-64	168 (20.9)		2998 (15.3)		—	
	65-74	253 (31.5)		6470 (33.0)		—	
	≥75	333 (41.4)		9600 (49.0)		—	
**Race, n (%)**							0.02
	American Indian or Alaska Native	15 (1.9)		275 (2.6)		—	
	Asian	20 (2.5)		765 (3.9)		—	
	Black or African American	79 (9.8)		2011 (10.3)		—	
	Native Hawaiian or other Pacific Islander	28 (3.5)		503 (2.6)		—	
	Unknown/Missing	87 (10.8)		1979 (10.1)		—	
	White	574 (71.4)		14,036 (71.7)		—	
**Ethnicity, n (%)**							0.01
	Hispanic or Latino	55 (6.8)		1569 (8.0)		—	
	Not Hispanic or Latino	695 (86.4)		16,837 (86.0)		—	
	Unknown/Missing	54 (6.7)		1177 (6.0)		—	
**Sex, n (%)**							–0.04
	Female	67 (8.3)		840 (4.3)		—	
	Male	737 (91.7)		18,743 (95.7)		—	
**Rurality, n (%)**							0.02
	Urban	592 (73.6)		14,574 (74.4)		—	
	Rural	197 (24.5)		4745 (24.2)		—	
	Highly Rural	8 (1.0)		191 (1.0)		—	
	Missing	7 (0.9)		74 (0.4)		—	
**Enrollment priority, n (%)**							0.01
	No special priority	113 (14.1)		2650 (13.5)		—	
	Low/moderate disability	137 (17.0)		3644 (18.6)		—	
	High disability	387 (48.1)		9246 (47.2)		—	
	Low income	165 (20.5)		3941 (20.1)		—	
	Missing	2 (0.2)		102 (0.5)		—	
**Diagnoses, n (%)**							
	Atrial fibrillation or flutter	316 (39.3)		6300 (32.2)		0.03	
	Heart failure	149 (18.5)		3405 (17.4)		0.01	
	Ischemic heart disease	256 (31.8)		8137 (41.6)		–0.04	
	Valvular heart disease	158 (19.7)		2530 (12.9)		0.04	
	Other diagnosis	205 (25.5)		5393 (27.5)		–0.01	

^a^CRH: Clinical Resource Hub.

^b^Standardized mean differences calculated through Cramer V for categorical variables and through Cohen *d* for continuous variables and binary categorical variables. Both were calculated in Stata 18.

^c^Indicates use of video care during the study period.

^d^Not applicable.

### Encounters

**Figure 2:**
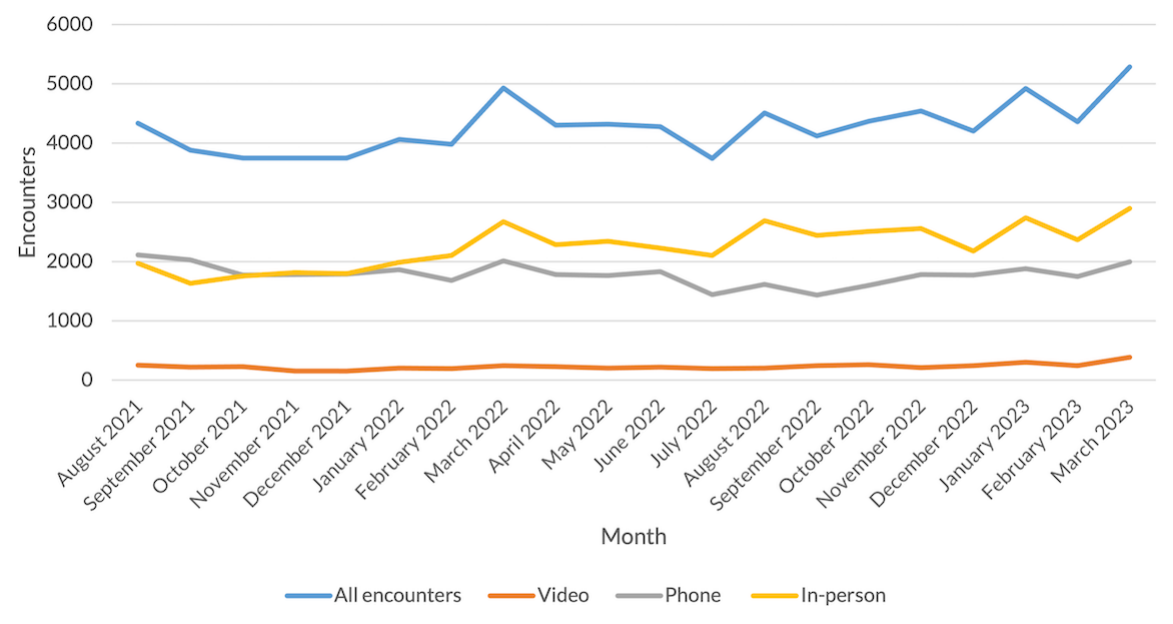
Cardiology encounters over time by encounter modality, Veterans Health Administration Sierra Pacific Region.

The total number of ambulatory cardiology encounters in the VA Sierra Pacific region remained approximately constant over the study period, including all CRH and non-CRH VA-based encounters (Figure 2). A slight uptick in total encounters took place in the first quarter of 2023. This was mostly due to an increase of 762 encounters (n=8003 vs n=7241; 11% growth) in in-person encounters compared with the fourth quarter of 2022, though telephone and video visits also increased over this period by 474 visits (n=5630 vs n=5156; 9% growth) and 224 visits (n=937 vs n=713; 31% growth), respectively.

**Figure 3:**
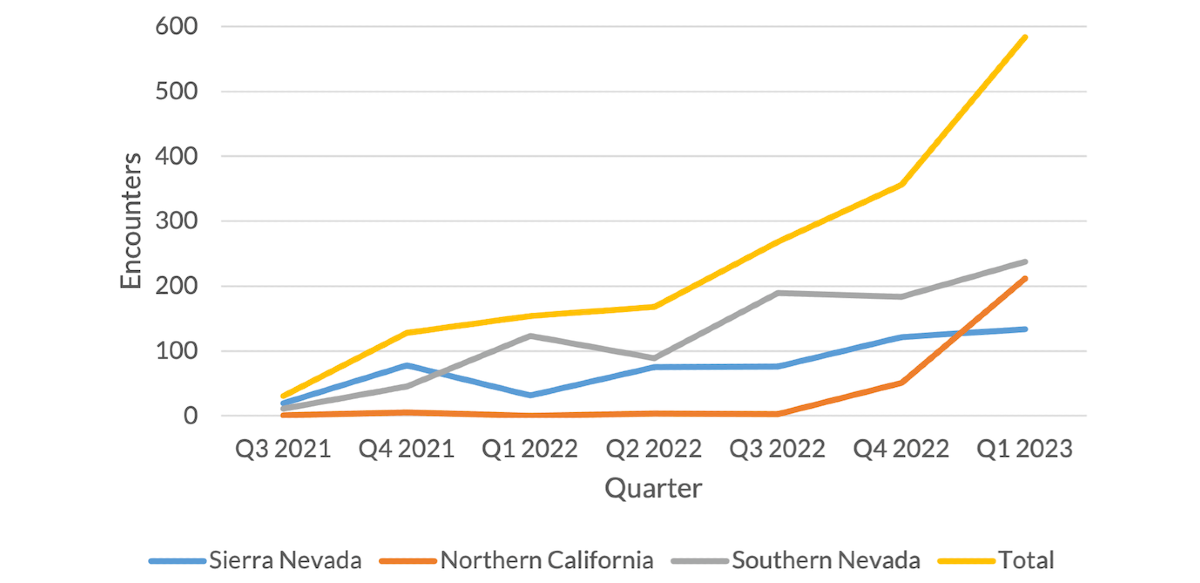
Clinical Resource Hub cardiology encounters over time.

Figure 3 shows the growth in CRH cardiology encounters over time at the 3 sites with the most CRH encounters. These curves closely reflect patient-level trends over time at these sites, with 33% (n=356 vs n=268) growth in the number of quarterly CRH encounters between the third and fourth quarters of 2022 and 64% (n=584 vs n=356) growth between the fourth quarter of 2022 and the first quarter of 2023. By Q1 2023, CRH encounters comprised approximately 3.5% of regional cardiology encounters. All CRH sites depicted increased their encounters substantially between the fourth quarter of 2022 and the first quarter of 2023, but at different rates: encounters for patients based at the Sierra Nevada site increased by 11% (n=134 vs n=121) over this period, whereas encounters for patients based at the Northern California site increased more than 4-fold over the same period from 51 to 212.

A total of 714 (36.4%) of the 1961 CRH encounters were conducted through video, with the remainder (1247/1961, 63.6%) conducted through telephone. For non-CRH encounters, 4.6% (3830/83,489) were conducted through video; 41.6% (34,757/83,489) through telephone; and just over half, or 53.8% (44,902/83,489), occurred in person.

### Adjusted Analyses

**Figure 4:**
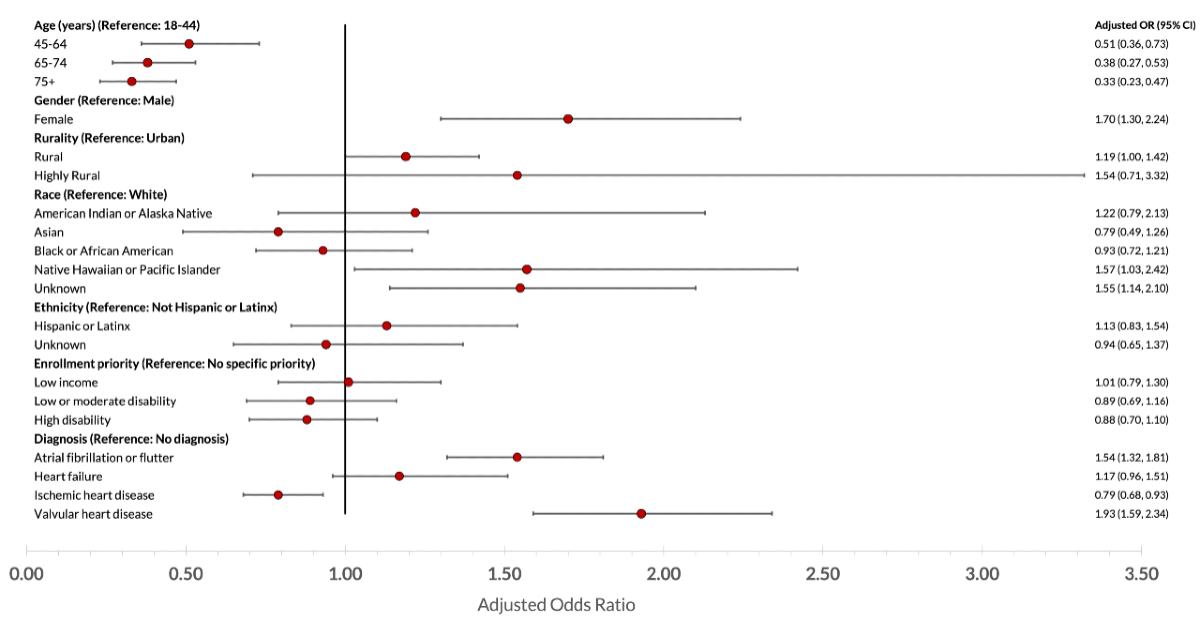
Adjusted odds of being a Clinical Resource Hub cardiology user. OR: odds ratio.

In our main multivariable logistic model, adjusted odds of using CRH were lower for older veterans (aged ≥75 years; adjusted odds ratio [OR] 0.33, 95% CI 0.23-0.47) and higher for female veterans (adjusted OR 1.70, 95% CI 1.30-2.24; Figure 4). Rural veterans had higher adjusted odds of using CRH (adjusted OR 1.19, 95% CI 1.00-1.42; *P*=.046) compared with the reference group of urban veterans, although the 95% CI for rural veterans overlapped to a great degree with the CI for highly rural and missing-rurality veterans. There were few significant differences by race or ethnicity, although patients of Native Hawaiian or other Pacific Islander race had higher odds of using CRH (adjusted OR 1.57, 95% CI 1.02-2.42). The enrollment priority group was not significantly associated with CRH use. Having a diagnosis of atrial fibrillation or flutter or valvular heart disease was associated with higher adjusted odds of using CRH (adjusted OR 1.54, 95% CI 1.32-1.81 and adjusted OR 1.93, 95% CI 1.59-2.34, respectively), whereas a diagnosis of ischemic heart disease was associated with lower odds (adjusted OR 0.79, 95% CI 0.68-0.93). Missing or unknown rurality was not pictured, which had an adjusted OR of 3.48 (95% CI 1.37-8.82).

Table S2 in Multimedia Appendix 1 presents marginal probabilities of ever using CRH. These marginal probabilities varied for age group from 0.03 (95% CI 0.03-0.04) for veterans aged 75 years or older to 0.09 (95% CI 0.07-0.11) for those aged 18-44 tears. Female veterans’ marginal probability of using CRH was 0.06 (95% CI 0.05-0.07), compared with 0.04 (95% CI 0.04-0.04) for male veterans. The marginal probability of using CRH was higher for veterans with valvular heart disease, at 0.06 (95% CI 0.05-0.07), than without (0.04, 95% CI 0.03-0.04). For other characteristics, there were no significant differences in marginal probabilities.

In our secondary analysis assessing the association of ever using CRH and visit modality, ever using CRH was associated with much higher adjusted odds of ever using video care (adjusted OR 29.73, 95% CI 24.43-36.18; refer to Table 2). Age was associated with lower adjusted odds of video care use according to a gradient, with an adjusted OR of 0.44 (95% CI 0.33-0.58) for veterans aged 75 years or older. Living in a rural location was associated with higher adjusted odds of video care use (adjusted OR 1.27, 95% CI 1.13-1.42), although this finding was not significant for those in highly rural locations (adjusted OR 1.30, 95% CI 0.83-2.03). Asian and Black or African American veterans had lower odds of video care use compared with White veterans (adjusted OR 0.68, 95% CI 0.50-0.91 and 0.78, 95% CI 0.65-0.93, respectively).

Marginal probabilities of video care use were higher for CRH users than nonusers at 0.64 (95% CI 0.62-0.68) and 0.08 (95% CI 0.08-0.09), respectively (Table S3 in Multimedia Appendix 1). Marginal probabilities for video care use varied significantly by age, from 0.09 (95% CI 0.08-0.09) for veterans aged 75 years or older to 0.16 (95% CI 0.13-0.19) for those aged 18-44 years. This figure was slightly higher for rural veterans than urban dwellers (0.12, 95% CI 0.11-0.12 for rural veterans compared to 0.10, 95% CI 0.09-0.10 for urban veterans). The marginal probability of using video care was significantly higher for veterans with valvular heart disease, at 0.14 (95% CI 0.13-0.15), than without (0.10, 95% CI 0.09-0.10). For other characteristics, there were no significant differences in marginal probabilities.

**Table 2:** Adjusted odds of using video care during the study period among all cohort patients (N=20,387).

		Adjusted odds ratio (95% CI)
**CRH^a^** **user status**		
	Never used CRH	Reference category
	Used CRH	29.73 (24.43-36.18)
**Age group (years)**		
	18-44	Reference category
	45-64	0.80 (0.61-1.06)
	65-74	0.57 (0.43-0.75)
	≥75	0.44 (0.33-0.58)
**Race**		
	White	Reference category
	American Indian or Alaska Native	1.27 (0.85-1.88)
	Asian	0.68 (0.50-0.91)
	Black or African American	0.78 (0.65-0.93)
	Native Hawaiian or other Pacific Islander	0.84 (0.58-1.20)
	Unknown or Missing	0.90 (0.74-1.09)
**Ethnicity**		
	Not Hispanic or Latino	Reference category
	Hispanic or Latino	0.91 (0.75-1.10)
	Unknown or Missing	1.12 (0.89-1.42)
**Sex**		
	Male	Reference category
	Female	1.20 (0.96-1.50)
**Rurality**		
	Urban	Reference category
	Rural	1.27 (1.13-1.42)
	Highly Rural	1.30 (0.83-2.03)
	Missing	1.81 (0.74-4.46)
**Enrollment priority**		
	No special priority	Reference category
	Low or moderate disability	1.03 (0.86-1.23)
	High disability	1.13 (0.97-1.31)
	Low income	0.81 (0.68-0.97)
	Missing	0.54 (0.23-1.24)
**Diagnoses**		
	Atrial fibrillation or flutter	1.23 (1.10-1.38)
	Heart failure	1.37 (1.21-1.55)
	Ischemic heart disease	1.25 (1.13-1.39)
	Valvular heart disease	1.69 (1.48-1.94)

^a^CRH: Clinical Resource Hub.

## Discussion

### Principal Findings

The VA has implemented this regionalized hub-and-spoke, primarily telehealth cardiology clinic to extend specialty care services to individuals in low-access communities across the region. In VA’s Sierra Pacific region, the CRH program served 800 veterans hailing from across the region in nearly 2000 telehealth encounters for evaluation and management of cardiovascular disease in its first 2 years of operation, and numbers of both patients and encounters increased at a steady-to-increasing rate. The CRH program reached women and rural-dwelling veterans at higher rates and highly disabled and low-income veterans at similar proportions compared with conventional VA-based cardiology clinics in the same region; conversely, the CRH patient population skewed younger than the conventional VA clinic population. This suggests that such a predominately telehealth, regionalized model of specialty care may be an effective method for accessing care for many high-need groups, although more targeted efforts may be required to reach older individuals. As more than half of rural-dwelling Americans live more than 20 km from the nearest cardiologist, and 95% live more than 20 km from a heart failure specialist [8], exploring telehealth-predominant care models like the CRH to expand access to these specialists is a priority both within and beyond the VA.

The reporting of these early results coincides with a shift in telehealth use from effectively a requirement during the national emergency phase of the COVID-19 pandemic, to an option for patients and clinicians alike [17]. This evolution brings both an opportunity and a mandate for rigorous study of how, when, and for whom telehealth should be used, and how telehealth visits affect the quality of care, resource use, and health outcomes [18]. This study is formative, with a focus on examining patterns of use; this lays a foundation for follow-up studies delving into the latter set of questions.

The concern of the digital divide [19] is ever present when considering the use of telehealth: will a primarily telehealth-based model of care inadvertently exclude groups frequently falling on the wrong side of the divide, such as those who are rural dwelling or have low income? Based on these findings, this particular program has reached historically marginalized groups in VA, such as women, racial or ethnic minority veterans, or those who are highly disabled or have low income, at similar or higher rates than the conventional model. A notable exception is among older individuals, who used CRH at much lower rates than their younger counterparts. The majority of older individuals in the United States are interested in conducting visits via telehealth [20], yet disparities in use by age have been widely demonstrated in VA both in general and in cardiology [9,10]. Establishing the source or sources of this discrepancy—whether due to true differences in interest in receiving care via a primary telehealth model, lower rates of offering the CRH program to older individuals, familiarity with navigating telehealth technologies, or other factors—will be an important focus of follow-up work.

We found that patients with diagnoses of atrial fibrillation/flutter or valvular heart disease had higher adjusted odds of being CRH users, unlike patients with diagnoses of heart failure or ischemic heart disease. This finding may reflect program-specific offerings (for example, clinics or physicians in the hub site with particular expertise in managing these conditions, or a perceived lack of capacity to manage them at spoke sites), or a sense that these conditions are more amenable to primarily telehealth management. Planned qualitative work, including interviews with program clinicians and administrators, will help to differentiate between these possible drivers.

For telehealth models designed to improve patient access to a given service, it is essential to establish whether that model offloads the conventional model, as intended, or simply induces more demand (eg, patients whose cardiovascular diseases would have otherwise been cared for in a primary care setting are instead referred for cardiology care). While this study does not aim to definitively answer this question, the fact that total cardiology encounters remained constant in the region over the study period suggests that there was not a strong demand-creation effect of the CRH model. However, to date, CRH patients comprise only a small fraction of total regional patients using cardiology services, so continued attention to this question will be important as the program grows.

### Limitations

Within the current data and study design, we are limited in the interpretation of various aspects of our findings. For example, although we can capture which CRH users have also used conventional care, our data lack the granularity to understand how and when this is the case; subsequent qualitative work will further elucidate these care patterns. At present, our data are limited to encounters within VA and do not extend to VA-purchased care in the community, meaning we cannot fully conclude whether CRH affects consumption of this costly form of care. This question, and characterization of other important facets of care associated with the program, such as patient, caregiver, and clinician satisfaction, clinical outcomes, and drivers of more and less successful program implementation, are left for future work. Finally, this analysis focused on a particular region and health care system and, therefore, may not be fully generalizable to other health care settings, although as Burnett et al [11] note in their publication on the early CRH implementation experience that “...some CRH design elements and experiences are unique to the [VA] system, [but] overall experience with telehealth hubs—including attempts to improve capacity for service provision, increase access, and deployment of telehealth services—is likely highly relevant to other health care systems.” In particular, the patient population in the VA includes fewer women and skews older than the general US population. We do note that female and younger veterans represent 2 subpopulations that used this program at disproportionately high rates compared to male and older veterans, respectively.

### Conclusions

The cardiology CRH program represents a telehealth-predominant, regionalized model of care that has extended specialty cardiology services to approximately 800 patients with low access to date in the VA region serving Northern California, Nevada, and the Pacific Islands. These data from the first 2 years of program implementation suggest that the program reached many of the most historically marginalized subpopulations of veterans, including female, rural-dwelling, and low-income veterans, at similar or higher rates compared with conventional cardiology care in the region. A notable exception was older individuals, who used CRH care at much lower rates; further work will examine the extent to which patient preference versus other factors drove this dynamic.

Many Americans live long distances from cardiologists, particularly in rural areas, prompting calls for the exploration of telehealth interventions to improve access [8]. This program represents one example of a regionalized, telehealth-predominant model that could be replicated in other regions and health care systems to address this widespread need.

## Appendix

Multimedia Appendix 1 International Classification of Diseases, 10th Revision codes for cardiovascular disease groups, and marginal probabilities of ever using Clinical Resource Hub and video care.

## Abbreviations

CRH: Clinical Resource Hub
ICD-10: International Classification of Diseases, 10th Revision
OR: odds ratio
VA: Veterans Health Administration

## Data Availability

The datasets generated during and/or analyzed during this study are not publicly available due to US Department of Veterans Affairs regulations and our ethics agreements, which do not permit data to leave the Veterans Health Administration (VA) firewall without a Data Use Agreement. This limitation is consistent with other studies based on VA data. However, VA data are made freely available to researchers with an approved VA study protocol. For more information, please contact the VA Information Resource Center at VIReC@va.gov.
