# Supplementary material for: Opportunities to Address Specialty Care Deserts and the Digital Divide through the Veterans Health Administration’s Telehealth Hub-and-Spoke Cardiology Clinic: Retrospective Cohort Study
Source: J Med Internet Res. 2024 Nov 28;26:e53932. doi: 10.2196/53932 (PMC11638694; doi:10.2196/53932)
Supplement: Multimedia Appendix 1 [file jmir_v26i1e53932_app1.docx]

Multimedia Appendix 1.

Table S1. ICD-10 Codes for Cardiovascular Disease Groups

Atrial fibrillation/flutter

I48.0 I48.11 I48.19 I48.20 I48.21 I48.3 I48.4

I48.91 I48.92

Heart failure (combined with cardiomyopathies)

I42.X I43

I50 I501 I502 I5020 I5021 I5022 I5023

I503 I5030 I5031 I5032 I5033 I504 I5040

I5041 I5042 I5043 I508 I5081 I50810 I50811

I50812 I50813 I50814 I5082 I5083 I5084 I5089

I509 I0981 I110 I130 I132

Ischemic Heart Disease

I21.X I22.X I20.X I23.0 I23.1 I23.2 I23.3

I23.6 I23.7 I23.8 I24.X I25.X Z95.1 Z95.5

Z98.61

Valvular Heart Disease

A52.03 I05.X I06.X I07.X I08.0 I08.1 I08.2

I08.9 I09.1 I34.X I35.X I36.X I37.X I38

I39 Q23.0 Q23.1 Q23.2 Q23.3 Z95.2 Z95.3

Z95.4

Table S2. Marginal Probabilities of Ever Using CRH

|  | **Adjusted Odds Ratio** | **95% Confidence Interval** |
| --- | --- | --- |
|  | | |
| **Age, years, categorical** |  |  |
| 18-44 | 0.09 | (0.07, 0.11) |
| 45-64 | 0.05 | (0.04, 0.06) |
| 65-74 | 0.04 | (0.03, 0.04) |
| 75+ | 0.03 | (0.03, 0.04) |
| **Race** |  | |
| American Indian or Alaska Native | 0.05 | (0.02, 0.07) |
| Asian | 0.03 | (0.04, 0.05) |
| Black or African American | 0.04 | (0.03, 0.04) |
| Native Hawaiian or other Pacific Islander | 0.06 | (0.04, 0.08) |
| Unknown/Missing | 0.06 | (0.04, 0.07) |
| White | 0.04 | (0.03, 0.04) |
| **Ethnicity** |  | |
| Hispanic or Latino | 0.04 | (0.03, 0.06) |
| Not Hispanic or Latino | 0.04 | (0.04, 0.04) |
| Unknown/Missing | 0.04 | (0.03, 0.05) |
| **Sex** |  |  |
| Female | 0.06 | (0.05, 0.07) |
| Male | 0.04 | (0.04, 0.04) |
| **Rurality** |  | |
| Urban | 0.04 | (0.03, 0.04) |
| Rural | 0.04 | (0.04, 0.05) |
| Highly Rural | 0.06 | (0.02, 0.09) |
| Missing | 0.11 | (0.03, 0.18) |
| **Enrollment Priority** |  | |
| No special priority | 0.04 | (0.04, 0.05) |
| Low/moderate disability | 0.04 | (0.03, 0.04) |
| High disability | 0.04 | (0.03, 0.04) |
| Low income | 0.04 | (0.04, 0.05) |
| Missing | 0.03 | (-0.01, 0,06) |
| **Diagnoses** |  | |
| No Atrial Fibrillation/Flutter | 0.03 | (0.03, 0.04) |
| Atrial Fibrillation/Flutter | 0.05 | (0.05, 0.06) |
| No Heart Failure | 0.04 | (0.04, 0.04) |
| Heart Failure | 0.04 | (0.03, 0.05) |
| No Ischemic Heart Disease | 0.04 | (0.04, 0.05) |
| Ischemic Heart Disease | 0.03 | (0.03, 0.04) |
| No Valvular Heart Disease | 0.04 | (0.03, 0.04) |
| Valvular Heart Disease | 0.06 | (0.05, 0.07) |

Table S3. Marginal Probabilities of Ever Using Video Care

|  | **Margin** | **95% Confidence Interval** |
| --- | --- | --- |
|  | | |
| **CRH Non-User** | 0.08 | (0.08, 0.09) |
| **CRH User** | 0.64 | (0.62, 0.68) |
| **Age, years, categorical** |  | |
| 18-44 | 0.16 | (0.13, 0.19) |
| 45-64 | 0.14 | (0.12, 0.15) |
| 65-74 | 0.11 | (0.10, 0.11) |
| 75+ | 0.09 | (0.08, 0.09) |
| **Race** |  | |
| American Indian or Alaska Native | 0.13 | (0.09, 0.16) |
| Asian | 0.08 | (0.06, 0.10) |
| Black or African American | 0.09 | (0.08. 0.10) |
| Native Hawaiian or other Pacific Islander | 0.09 | (0.07, 0.12) |
| Unknown/Missing | 0.10 | (0.08, 0.11) |
| White | 0.11 | (0.10, 0.11) |
| **Ethnicity** |  | |
| Hispanic or Latino | 0.09 | (0.08, 0.11) |
| Not Hispanic or Latino | 0.10 | (0.10, 0.11) |
| Unknown/Missing | 0.11 | (0.09, 0.13) |
| **Sex** |  |  |
| Female | 0.12 | (0.10, 0.13) |
| Male | 0.10 | (0.10, 0.10) |
| **Rurality** |  | |
| Urban | 0.10 | (0.09, 0.10) |
| Rural | 0.12 | (0.11, 0.12) |
| Highly Rural | 0.12 | (0.08, 0.16) |
| Missing | 0.15 | (0.05, 0.25) |
| **Enrollment Priority** |  | |
| No special priority | 0.10 | (0.09, 0.11) |
| Low/moderate disability | 0.10 | (0.09, 0.11) |
| High disability | 0.11 | (0.10, 0.12) |
| Low income | 0.08 | (0.08, 0.09) |
| Missing | 0.06 | (0.02, 0.10) |
| **Diagnoses** |  | |
| No Atrial Fibrillation/Flutter | 0.10 | (0.09, 0.10) |
| Atrial Fibrillation/Flutter | 0.11 | (0.11, 0.12) |
| No Heart Failure | 0.10 | (0.09, 0.10) |
| Heart Failure | 0.12 | (0.11, 0.13) |
| No Ischemic Heart Disease | 0.09 | (0.09, 0.10) |
| Ischemic Heart Disease | 0.11 | (0.11, 0.12) |
| No Valvular Heart Disease | 0.10 | (0.09, 0.10) |
| Valvular Heart Disease | 0.14 | (0.13, 0.15) |
